# Accurate, affordable, and easy electrochemical detection of ascorbic acid in fresh fruit juices and pharmaceutical samples using an electroactive gelatin sulfonamide

**DOI:** 10.1039/d4ra06271j

**Published:** 2024-12-17

**Authors:** Hend S. Magar, Asmaa M. Fahim, M. S. Hashem

**Affiliations:** a Applied Organic Chemistry Department, National Research Centre Dokki, P. O. Box. 12622 Giza Egypt hendamer2000@yahoo.com; b Department of Green Chemistry, National Research Centre Dokki, P. O. Box. 12622 Giza Egypt; c Polymers and Pigments Department, National Research Centre Dokki, P. O. Box. 12622 Giza Egypt ms.hashem@nrc.sci.eg

## Abstract

In this study, we demonstrated how to design and construct a highly specific and sensitive sensor capable of rapidly and accurately detecting ascorbic acid (AA). A sulfonamide derivative (S) acting as a novel monomer was synthesized through an aldol condensation reaction. Subsequently, a free radical-mediated grafting polymerization approach was used to create a new generation of gelatin (Gel) grafted with poly sulfonamide derivative (Gel-g-PS). The graft percentage (GP%) was 60 ± 0.5% with a conversion rate of 98.3%. Fourier-transform infrared spectroscopy (FT-IR) and scanning electron microscopy (SEM) were utilized to confirm the formation of Gel-g-PS. The developed gelatin sulfonamide modified screen printed electrode (Gel-g-PS/SPE) was employed for the determination of ascorbic acid (AA) in fruit juices and pharmaceutical samples. Gel-g-PS/SPE showed excellent electrochemical catalytic activities toward AA oxidation compared to bare (unmodified) SPE. Ascorbic acid displayed a sensitive oxidation peak at 0.35 V using the differential pulse voltammetry technique. Under optimized experimental conditions, the two linear ranges for AA detection were obtained to be from 0.2–5 ppb and 20–600 ppb, with a limit of detection (LoD) of 0.03 ppb and a limit of quantification (LoQ) of 0.11 ppb. The proposed Gel-g-PS modified SPE surface demonstrated good selectivity, stability, reproducibility, and repeatability as well as a good recovery rate in fresh fruit juices and pharmaceutical samples.

## Abstract 2

Synthesis of electroactive gelatin grafted with poly sulfonamide derivative (Gel-g-PS) *via* a free radical-mediated grafting polymerization for a highly selective and sensitive electrochemical sensor for detecting ascorbic acid (AA) in real samples.

## Graphical abstract

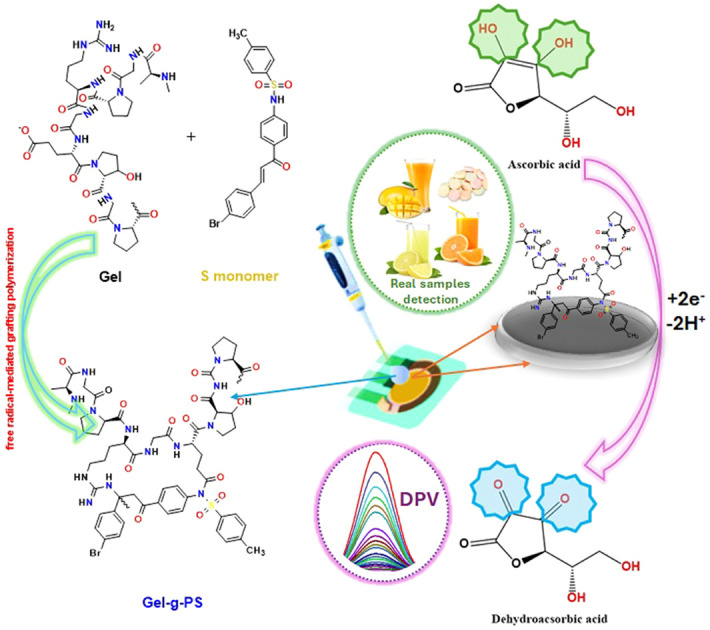

## Introduction

Gelatin is a naturally occurring protein with a three-dimensional triple helix structure, derived from collagen found in animal bones, connective tissues, and skin. It is known for its biocompatibility, biodegradability, biosafety, affordability, high dispersibility, and sol–gel characteristics. Gelatin has numerous reaction sites due to its active groups, including amino, carboxylic, and hydroxyl. Because of these qualities, gelatin and its derivatives are considered ideal materials for creating sensors.^1^ Gelatin-containing sensors have become increasingly popular in the medical diagnostic, food testing,^2^ and ecological monitoring industries in recent years.^3–5^

Vitamins are organic compounds required for metabolism that the human body is unable to produce in sufficient amounts, so they must be obtained through diet.^6^ Ascorbic acid (AA), vitamin C, is found in various fruits and vegetables and widely used as an antioxidant in food and pharmacological compositions, as well as a reducing agent and enzyme co-factor in the human body's metabolic process.^7–9^ It plays a crucial role in many important human life processes as it is essential for encouraging the production of antibodies, iron absorption, and serving as a preventative factor for oxidative effects on cells and tissues.^10,11^ An ascorbic acid deficiency can lead to illnesses such as anemia, cancer, colds, hypertension, mental disorders, deterioration of neurotransmitters, and scurvy.^12,13^ Therefore, developing accurate, reliable, rapid and easy-to-implement methods for measuring low levels of ascorbic acid in real samples is of great importance for the clinical evaluation of pertinent illnesses.^14,15^ Even though AA is unstable in aqueous solutions since it is easily oxidized reversibly to dehydroascorbic acid and subsequently irreversibly to 2,3-diketo-l-gulonic acid, making it difficult to quantify. Several methods have been developed for its detection like enzymatic,^16^ titrimetric,^17^ fluorometric,^18^ spectrophotometric,^19^ chromatographic,^20^ and electrochemical approaches.^21^ Electrochemical sensors^22–24^ have attracted much interest because their accuracy,^25–27^ cost-effectively^28,29^ simplicity,^30^ super-sensitivity^31^ with reliability,^32^ rapid response,^33–35^ electrode stability,^36,37^ less sensitive towards the matrix effects than other analytical techniques, and needless for derivatization of the analytes.^38^ It is challenging to detect ascorbic acid directly using traditional electrodes because of high over potential, electrodes contamination, low selectivity, and inconsistent results.^39^ Researchers are exploring precise, selective electrodes made of modified materials for AA sensing in order to get around these problems. Common instances comprised carbon dots,^40^ metal nanoparticles, metal–organic frameworks,^41^ and polymers, *etc*. It is crucial to develop a portable, quick, and extremely sensitive real-time system for precise point-of-care AA detection because, although AA electrochemical detectors accomplish better when using polymeric materials, they have not yet been successfully confirming into commercial products.

Herein, we synthesized a novel monomeric material through an aldol condensation reaction based on a sulfonamide derivative. Then we created a gelatin grafted with a poly sulfonamide derivative hydrogel using a free radical-mediated grafting polymerization approach. A modified screen-printed electrode based on the inventive polymeric hydrogel, was used to develop an electrochemical ascorbic acid sensor that is both selective and sensitive. The developed electrochemical sensor will be applied in the development of portable point-of-care testing systems with a sample-in, answer-out methodology that allows for use by non-specialized personnel. Screen-printed electrodes were chosen because they enable measurements in a minimal sample volume. The gelatin sulfonamide modified electrode has shown high electro-catalytic activity toward ascorbic acid detection, as demonstrated in studies using differential pulse voltammetry (DPV) technique. The voltammetry curves showed superior analytical capabilities and a high degree of selectivity for ascorbic acid.

## Materials and methods

Gelatin (Gel) was purchased from Aldrich, UK. The novel sulfonamide derivative monomer, (*E*)-*N*-(4-(3-(4-bromophenyl) acryloyl) phenyl)-4-methyl benzene sulfonamide (S), was synthesized and characterized as previously described.^42^ Potassium peroxydisulphate (PPS) was obtained from BDH Laboratory Supplies in Poole, England. Potassium chloride (KCl), potassium ferricyanide (K_3_[Fe(CN)_6_]), potassium ferrocyanide (K_4_[Fe(CN)_6_]·3H_2_O), ascorbic acid (AA), uric acid, dopamine, sodium chloride (NaCl), sodium nitrate (NaNO_3_), magnesium nitrate (Mg(NO_3_)_2_), potassium nitrate (KNO_3_), sodium hydroxide (NaOH), sodium dihydrogen phosphate (NaH_2_PO_4_), disodium hydrogen phosphate (Na_2_HPO_4_), and sulfuric acid (H_2_SO_4_) were purchased from Sigma-Aldrich, Germany. All solutions were prepared either in a double-distilled water (DDW) or a 0.1 M phosphate-buffered solution (PBS, pH 7.4) unless otherwise specified. The supporting electrolyte, a 0.1 M PBS was created by mixing NaH_2_PO_4_ and Na_2_HPO_4_ in DDW. The pH of the solution was adjusted to fall within the range of 5 to 8 using H_2_SO_4_ and NaOH.

The chemical bonding of the polymer produced was examined using a Shimadzu 8101 Fourier-transform infrared spectrometer (FT-IR), which operates within the range of 400–4000 cm^−1^. Texture images were taken using a JEOL-JSM-6390LV device for scanning electron microscopy (SEM) supported by energy dispersive X-ray spectroscopy (EDX). A CHI 660 electrochemical workstation was used to record all cyclic and differential pulse voltammetric experiments (CV and DPV) and electrochemical impedance spectroscopy (EIS) measurements. Screen-printed electrodes (SPEs), incorporating working electrode, counter-electrode, and a silver pseudo reference electrode, were obtained from Orion Hi-Tech S. L. (Madrid, Spain) and were employed to perform all the electrochemical experiments.

### Synthesis of gelatin grafted with poly sulphonamide derivative (Gel-g-PS)

Gelatin sulfonamide was produced using our previous process with some modifications. In general, Gel-g-PS was synthesized through a free radical-mediated grafting polymerization approach with 0.15 g of PPS as the initiating agent. 1 g of Gel was heated at 40 °C with 80 ml of DDW up to completely dissolved. The gel solution was then mixed with 0.1 g of sulfonamide derivative monomer and ultrasonicated until fully suspended. The suspended mixture was added to the reaction container and aggressively stirred allowing polymerization for 2 hours at 65 °C. Residual Gel was subsequently rinsed with hot DDW, and the resulting hydrogel was submerged in ethanol for 1 week to remove any unaltered monomer and homopolymer. The produced hydrogel was allowed to dry for 1 day at 40 °C and then preserved at room temperature for additional characterizations.

### Graft percentage and conversion determination

The grafting parameters including the graft percentage (GP%) and conversion (%) of the synthesized Gel-g-PS were determined and calculating utilizing eqn (1) and (2).^43^1
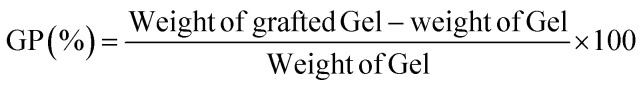
2
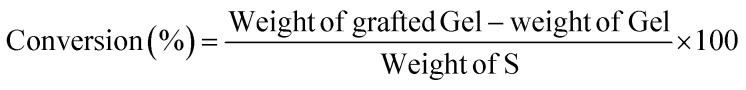


### Preparation of the modified electrode

For screen-printed electrode (SPE) modification, a homogenous suspended solution of Gel or Gel-g-PS in DDW (10 mg ml^−1^) were obtained after sonication for 30 minutes. Next, 10 μL of Gel-g-PS suspended solution was dropped onto the screen-printed electrode surface and left to dry at room temperature.

### Electrochemical measurements

CV and EIS electrochemical characterizations were obtained by using a solution containing 5 mM [Fe(CN)_6_]^4−/3−^ and 0.1 M PBS. In CV measurements, the potential ranged from −0.8 to +1.0 V with a scan rate of 50 mV s^−1^. EIS measurements utilized an amplitude modulation of −20 mV and a frequency range of 10^5^ Hz to 0.1 mHz. For ascorbic acid (AA) detection, CV, and DPV measurements were taken at various concentrations of AA with scan rate of 50 mV s^−1^. The potential range was −1.0 V to 1.0 V for CV measurements and 0 to 0.8 V for DPV detection. In the selectivity study, DPV measurements were taken for 120 ppb of different interferents (uric acid, dopamine, K^+^, Na^+^, Mg^2+^, Cl^−^, SO_4_^2−^, and NO_3_^−^) and 60 ppb of AA.

### Real sample preparation

Vitamin C tablets as well as fresh lemon, orange, and mango fruits were weighed and crushed well in a mortar. They were then dissolved in a PBS with sonication for 30 minutes, centrifuged at 5000 rpm for 4 minutes, and filtered using Whatman filter paper. Approximately 1 ml of the clear solution was diluted with 100 ml of PBS (pH 7.4) in a volumetric flask. A specific volume of the diluted solution was then transferred into an electrochemical cell.^44–46^

### Statistical analysis

The experiments were conducted in triplicate and the data was presented as means ± SD. Statistical analysis was performed using IBM SPSS Statistics 29.0.10 to compare the control and testing materials. A *P*-value of <0.05 was considered statistically significant.

## Results and discussion

### Characterization of gelatin grafted with poly sulfonamide derivative

**Fig. 1:**
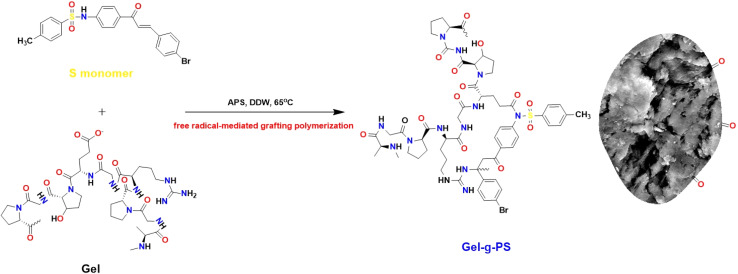
The schematic representation of Gel-g-PS formation.

This work used the free radical-mediated grafting polymerization approach of a sulfonamide derivative to onto a Gel backbone structure with PPS as a thermal initiating agent. The persulfate decomposed at 65 °C producing sulfate radicals that caused the formation of new active initiating sites on the Gel chains, leading to the start of the polymerization process of the sulfonamide derivative. Fig. 1 illustrated how a new novel monomer was polymerized to create a novel grafted polymeric material. Eqn (1) and (2) were used to calculate the grafting characteristics of the synthesized Gel-g-PS, specifically the graft percentage (GP%) and conversion (%). The graft percentage (GP %) was 60 ± 0.5% with a conversion of (%) 98.3%.

### Infrared specroscopy

**Fig. 2:**
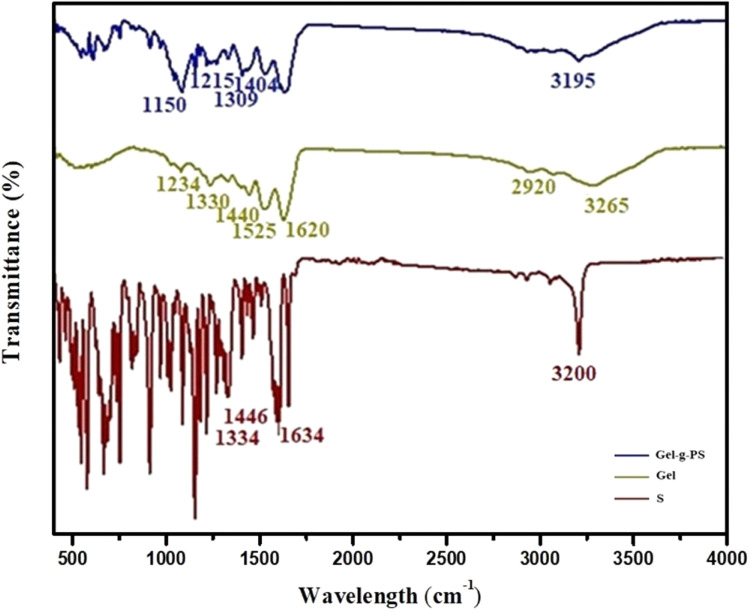
FT-IR spectra of S, Gel, and Gel-g-PS.

The interaction between molecules and their functional groups was explored through FT-IR in a wavenumber range between 400 and 4000 cm^−1^. Fig. 2 displayed FT-IR spectra of unmodified Gel, S, and Gel-g-PS. In unmodified Gel, the nitrogen-hydrogen stretching bond of the amino group residues, and the carboxylate group residues located at the same region leading to the appearance of a wide broad band around 3265 cm^−1^. The asymmetric stretching of methylene groups was observed around 2920 cm^−1^. The stretching carbonyl, bending nitrogen–hydrogen, and stretching carbon–nitrogen bonds existed around 1620, 1525 cm^−1^, and 1234 cm^−1^, respectively. The symmetric and asymmetric vibrational bending of the methyl groups were found around 1330 cm^−1^ and 1440 cm^−1^. In the spectra of Gel-g-PS, as shown in Fig. 2, there was a superposition between S novel monomeric peaks and the unmodified Gel. The symmetrical and asymmetrical stretching of sulfonamide group were around 1150 and 1215 cm^−1^ and 1309 and 1404 cm^−1^, respectively, which proved the grafting polymerization process of the poly sulfonamide derivative. The decrease in the strength of the sharp peak at 3200 of the nitrogen–hydrogen bonds in S was due to the interaction between amide and carboxylate group residue in S and Gel, respectively. The extensive absorption region features a strong peak at 3195 cm^−1^ which is connected to the intersection of the nitrogen hydrogen stretching and hydroxyl group vibrations of sulfonamide derivative and Gel, respectively. Also, the peak at 1625 cm^−1^ corresponds to the nitrogen–hydrogen bending vibration of amides of the sulfonamide derivative. The presence of characteristic peaks of the sulfonamide derivative composition along with the characteristic peaks of the gelatin indicates the grafting polymerization of the sulfonamide derivative onto the Gel backbone.

### Morphological surface structure

**Fig. 3:**
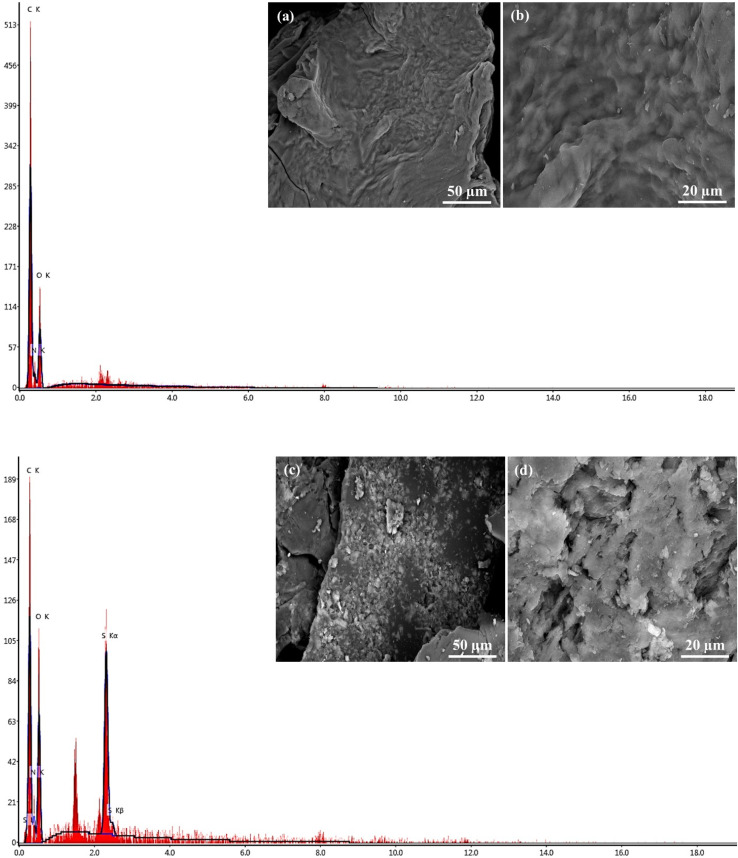
SEM images and EDX of (a and b) unmodified Gel and (c and d) Gel-g-PS.

The morphology of Gel-g-PS was investigated using scanning electron microscopy and energy dispersive X-ray spectroscopy. The unmodified Gel surface appears as crimpy layers with wavy ridges overlapping together as shown in Fig. 3a and b. After a free radical-mediated graft polymerization process, the texture of Gel-S consists of two layers: the first one is Gel enveloped with crushed granules of poly sulfonamide derivative as observed in Fig. 3c and d. These morphological changes affirmed that the poly sulfonamide derivative grafted onto the surface of the Gel structure. Additional evidence for the grating polymerization of the sulfonamide derivative is the EDX investigation, which confirmed the presence of sulfur.

### Electrochemical aspects

**Fig. 4:**
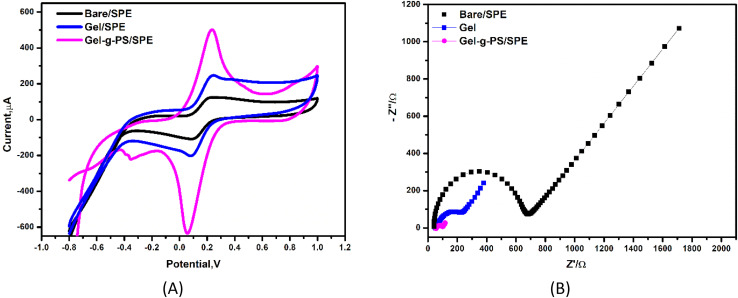
Electrochemical characterization. (A) Cyclic voltammetry curves and (B) electrochemical impedance spectroscopy (EIS) of bare (unmodified)/SPE, Gel/SPE and Gel-g-PS/SPE in a mixture solution of 5 mM of FCN redox probe and 0.1 M of KCl at scan rate of 50 mV s^−1^.

The use of electrochemical systems for tracking and monitoring the enhancement of the electron transport from redox solution toward electrode surface requires a strong sensing platform supported by conducting materials to provide highly catalytic activity and high conductivity. Therefore, a new generation of gelatin grafted with poly sulfonamide derivative (Gel-g-PS) were tested using cyclic voltammetry (CV) and electrochemical impedance spectroscopy (EIS) techniques. The electrochemical characterizations (CV & EIS) of bare (unmodified)/SPE, Gel/SPE and Gel-g-PS/SPE using the redox probe solution of [Fe(CN)_6_]^4−/3−^ were performed as shown in Fig. 4. In CV measurements, as seen in Fig. 4A and Table 1, the redox peak current values (*i*_pa_ and *i*_pc_) increased from bare/SPE (*i*_pa_ = 125.9 μA, *i*_pc_ = −110.3 μA) to Gel/SPE (*i*_pa_ = 249.5 μA, *i*_pc_ = −200.8 μA). The highest redox peak current values were observed for Gel-g-PS/SPE (*i*_pa_ = 510.6 μA, *i*_pc_ = −640.5 μA). Therefore, Gel-g-PS/SPE enhanced the transport of electrons from the redox solution towards the electrode surface. Additionally, in EIS measurements, as observed in Fig. 4B and Table 1, the change in the electrode surface was noted through the charge transfer resistance (*R*_ct_) values obtained after fitting the Nyquist EIS plot with a certain circuit. This circuit included the resistance of the solution (*R*_s_), charge transfer resistance (*R*_ct_), capacitance (*C*) and Warburg resistance (*W*). The lowest *R*_ct_ value was obtained for Gel-g-PS/SPE (*R*_ct_ = 62.5 Ω) compared to Gel/SPE (*R*_ct_ = 195.4 Ω) and Bare/SPE (*R*_ct_ = 625.8 Ω). Therefore, the Gel-g-PS modified SPE enhances the electrode surface, resulting in excellent electrical conductivity.

**Table 1:** The data which was evaluated from the CV and EIS experimental studies in Fig. 5

Electrode type	*I*_a_ (μA)	*I*_c_ (μA	*E*_oxd._ (V)	*E*_red._ (V)	*E*_1/2_ (V)	*R*_s_ (Ω)	*R*_ct (1)_ (Ω)	*W* (Ω)	*C* (F)
Bare/SPE (unmodified)	125.9	−110.3	0.259	0.094	0.176	39.2	625.8	0.0084	6.7 × 10^−6^
Gel/SPE	249.5	−200.8	0.237	0.076	0.153	40.6	195.4	0.0038	3.8 × 10^−5^
Gel-g-PS/SPE	510.6	−640.5	0.230	0.055	0.14	48.7	62.5	0.0013	6.15 × 10^−5^

### Scan rate effect

**Fig. 5:**
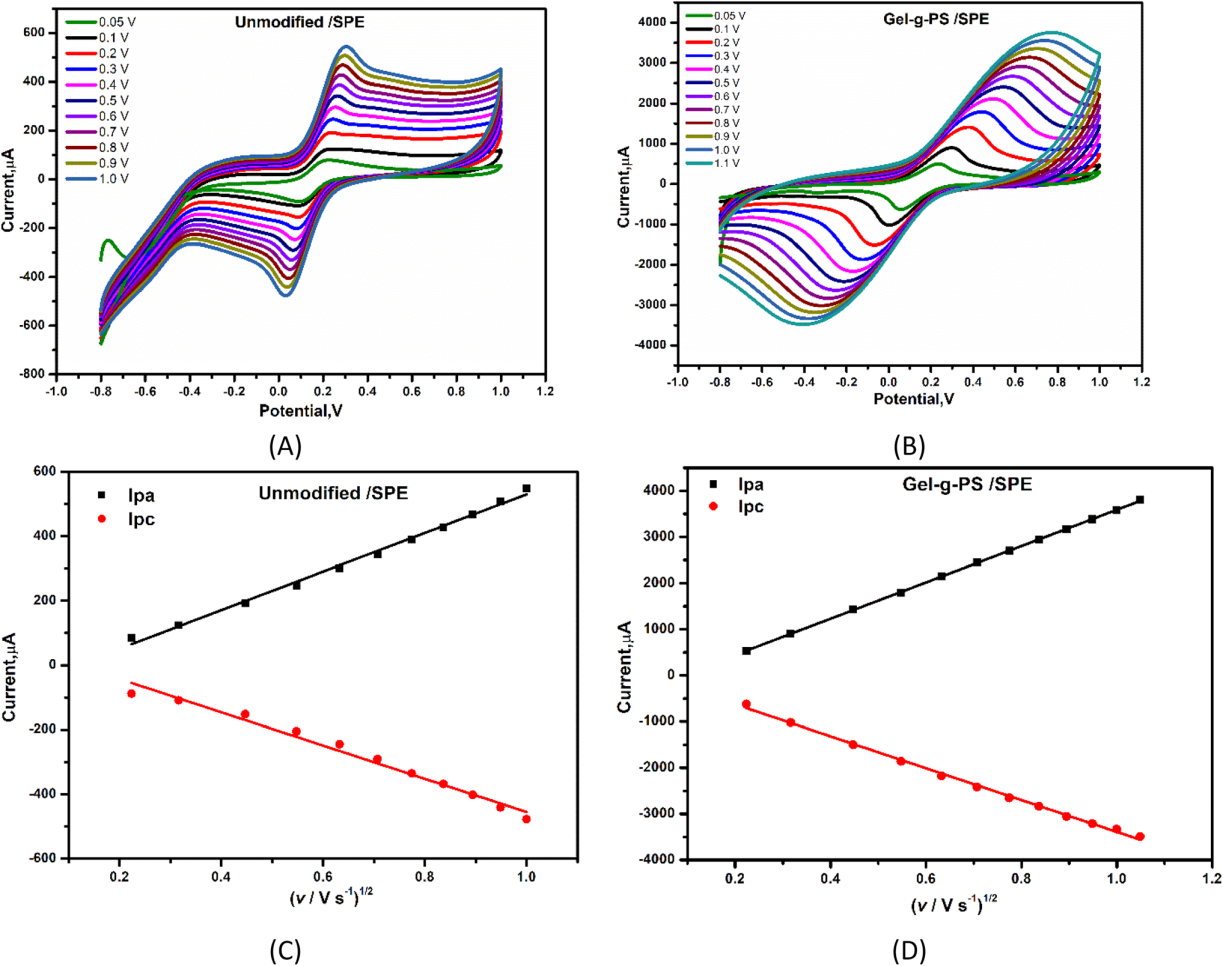
Oxidation and reduction peak currents of solution containing 5 mM [Fe(CN)_6_]^4−/3−^ and 0.1 M of KCl at different scan rates for (A) bare SPE (unmodified SPE) and (B) Gel-g-PS/SPE. *I*_pa_ and *I*_pc_ linearity lines corresponding to CVs for (C) bare, and (D) Gel-g-PS/SPE recorded at different scan rates.

To study the catalytic activity behavior and effective electrochemical active surface area (EASA) of the Gel-g-PS modified SPE, different scan rates with a range from 50 to 1000 mV s^−1^ for unmodified and Gel-g-PS modified SPE in a solution containing 5 mM [Fe(CN)_6_]^4−/3−^ and 0.1 M KCl were investigated using CV technique. Fig. 5A & B showed a linear increase in the current of the oxidation and reduction peaks of the FCN redox probe solution. Consequently, the Gel-g-PS modified SPE exhibited higher current readout activity compared to the unmodified bare/SPE. In Fig. 5C & D, the cathodic and anodic peak currents of FCN were plotted against the square root of the scan rate with linear lines for the unmodified bare/SPE regression equations of *I*_pa_ = 598.08*x* − 68.55 and *I*_pc_ = − 515.14*x* + 60.11, and correlation coefficient (*R*^2^) values of 0.997 and 0.994, respectively. For the Gel-g-PS/SPE, the regression equations were *I*_pa_ = 3931*x* − 340.7 and *I*_pc_ = −3455*x* + 64.22 with correlation coefficient (*R*^2^) values of 0.9996 and 0.9998, respectively. The effective electrochemical active surface area (EASA) of the unmodified and Gel-g-PS/SPE were calculated from Fig. 5C & D using the Randles–Sevcik eqn (3):

3 *I*_p_ = 2.69 × 10^5^ × *n*^3/2^ × *A* × *D*^1/2^ × *Cu*^1/2^

where the *I*_p_, *n*, *D*, *A*, *C* and *u* represented peak current in amperes, number of electron transferred, diffusion coefficient (cm^2^ s^−1^), electrochemical active area (cm^2^), concentration of the [Fe(CN)_6_]^3−/4−^ molecules (mol L^−1^), and scan rate (V s^−1^), respectively. *I*_pa_/*u*^1/2^ can be obtained from the slope value of Fig. 5C & D. The calculated active surface area for bare/SPE and Gel-g-PS/SPE were 0.0586 cm^2^, and 2.594 cm^2^, respectively. These values confirmed the enlargement of the Gel-g-PS/SPE surface area.

### Electrocatalytic activity effect of Gel-g-PS toward ascorbic acid response

To study the effect of Gel-g-PS electrocatalytic activity toward detecting ascorbic acid with high electron transfer efficiency, the sensitivity response of bare/SPE and Gel-g-PS/SPE toward ascorbic acid detection was examined by cyclic voltammetry in a solution containing PBS (pH 7.4) and a certain concentration of ascorbic acid (10^−2^ M) at a scan rate of 50 mV s^−1^.

**Fig. 6:**
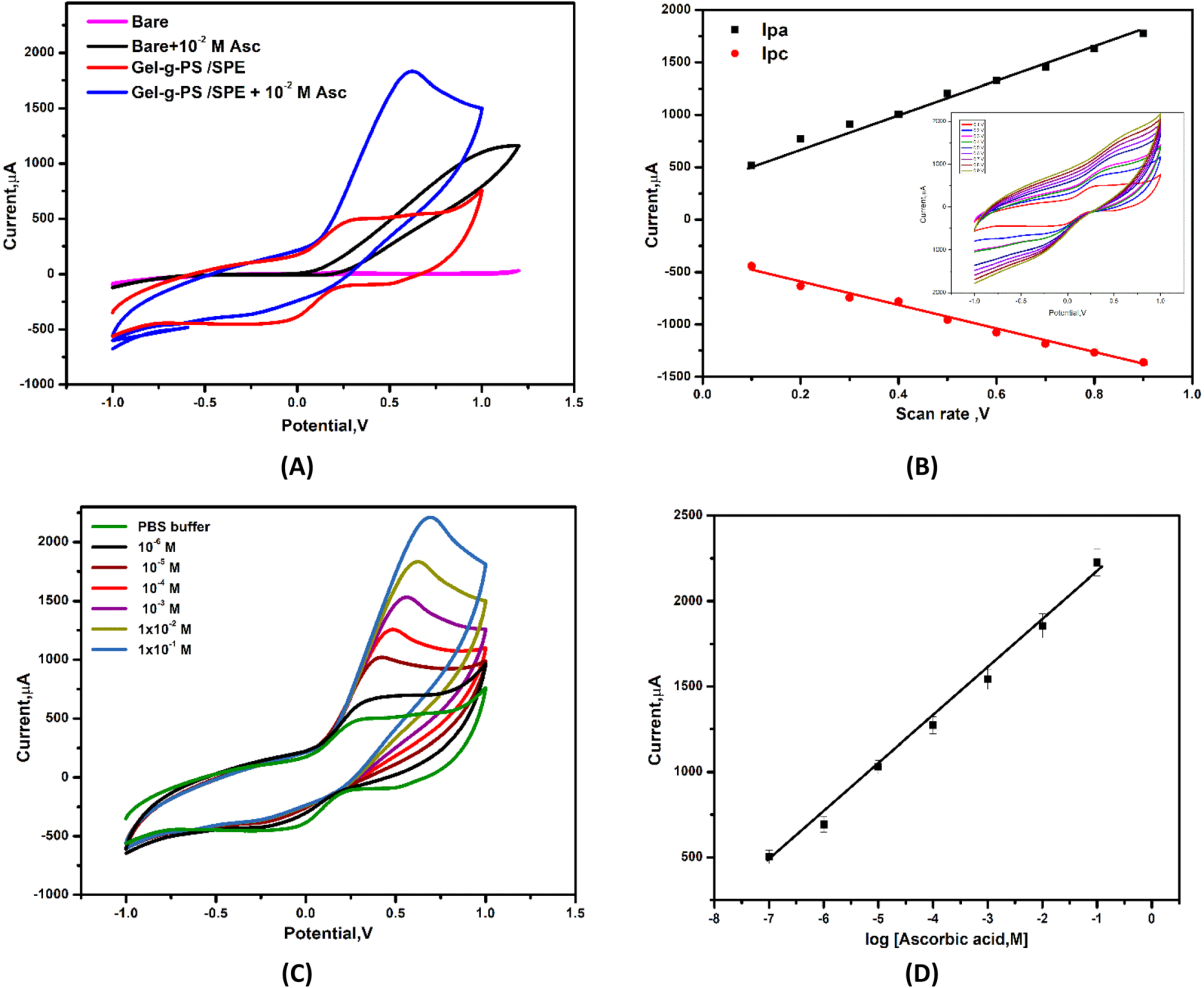
(A) Cyclic voltammograms of the bare/SPE and Gel-g-PS/SPE in PBS (pH 7.4) in the absence and present 10^−2^ M of ascorbic acid. (B) Scan rate effect of Gel-g-PS/SPE. (C) Cyclic voltammetry curves obtained after addition of different concentrations of ascorbic acid in PBS (pH 7.4). (D) Calibration curve of current *vs.* log concentration of AA.

From the cyclic voltammetry study of bare/SPE and Gel-g-PS/SPE within a potential range of −1.0 V to +1.0 V in a PBS (pH 7.4) as shown in Fig. 6A, Gel-g-PS/SPE exhibited a quasi-reversible CV peak due to the oxidation and reduction of Gel-g-PS. The oxidation occurs as the Gel-g-PS hydro-quinonoid form exchanges to the quinonoid form, with the reverse happening during reduction. Additionally, the current ratio of *I*_pc_/*I*_pa_ being close to unity suggests that the Gel-g-PS polymer maintained its reversibility even when deposited on the electrode's surface. The peak currents for reduction and oxidation increase linearly with an increase in scan rate value from 10 to 900 mV s^−1^, conforming the surface-confined electron transfer process and the deposition of the Gel-g-PS polymer on the SPE surface, as presented in Fig. 6B. Consequently, Gel-g-PS possessed advantageous electrochemical features for detecting ascorbic acid with high electron transfer efficiency.

**Fig. 7:**
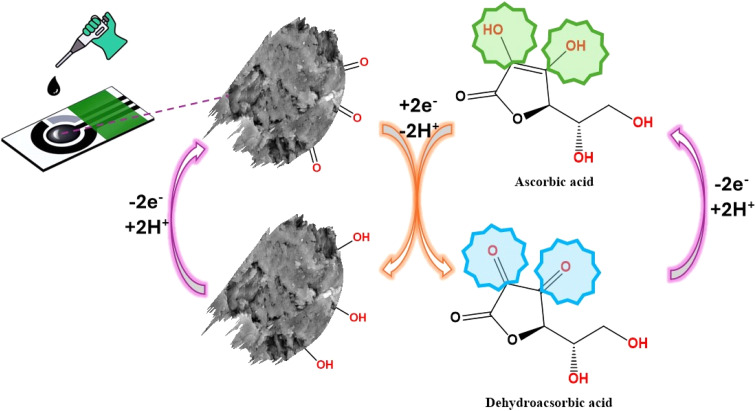
The schematic diagram showed the preparation and sensing response of Gel-g-PS -modified screen-printed electrode toward ascorbic acid detection.

Furthermore, increasing the concentration of AA caused an increase in the anodic peak current as shown in the cyclic voltammetry curves of different concentrations of AA in Fig. 7C and the calibration curve of various concentrations of ascorbic acid *vs.* anodic peak current with *R*^2^ = 0.992, as depicted in Fig. 6D. The sensing response mechanism of Gel-g-PS -modified screen-printed electrode toward ascorbic acid detection was represented in Fig. 7. The mechanism of the electrocatalytic reaction can be suggested by the following equations:

4 AA → dehydro-AA + 2H^+^ + 2e^−^

5 Gel-g-PS + 2H^+^ + 2e^−^ → H_2_-Gel-g-PS

6 H_2_-Gel-g-PS (electrode) → Gel-g-PS (electrode) + 2H^+^ + 2e^−^

### Sensor performances

**Fig. 8:**
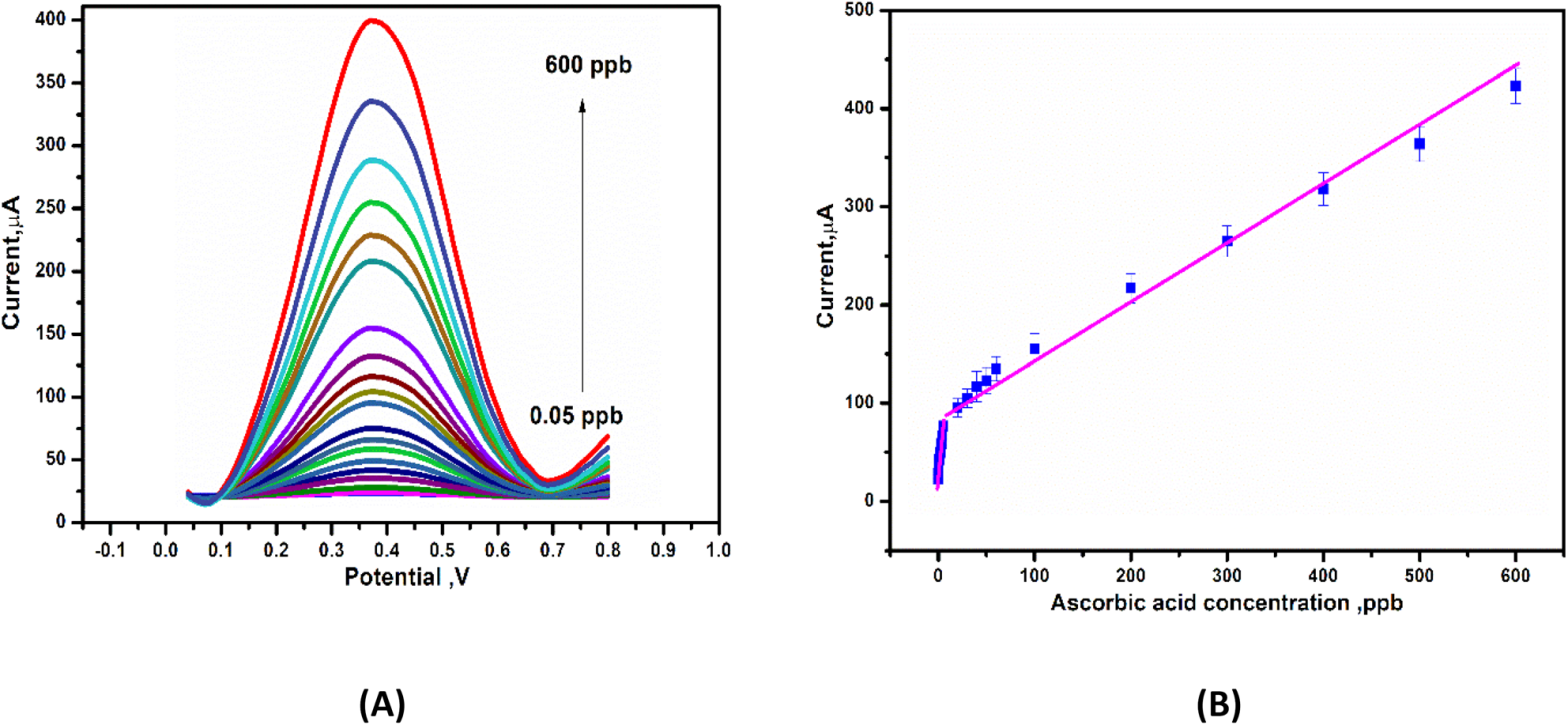
(A) Deferential pulse voltammetry (DPV) curves of Gel-g-PS/SPE in PBS (pH 7.4) containing different concentrations of ascorbic acid. (B) Calibration curve for ascorbic acid detection obtained from DPV measurements.

To develop a voltammetric methodology for detecting ascorbic acid, we selected the differential pulse voltammetry (DPV) mode, because the peaks were sharp and well-defined at lower concentrations of ascorbic acid. In DPV measurements, the anodic peak current increased with an increasing concentration of AA, as seen in Fig. 8A. This resulted in a linear relationship with two dynamic ranges from 0.2–5 ppb and 20–600 ppb, with slopes of 8.3 μA ppb^−1^ and 0.55 μA ppb^−1^ and *R*^2^ values of 0.98 and 0.977, respectively, as shown in Fig. 8B. The lower detection limit (LDL) was calculated using (3.3 × standard deviation)/slope to be 0.03 ppb and the limit of quantification (LoQ) was determined using (10 × standard deviation)/slope to be 0.11 ppb. Table 2 summarized some of the previously reported sensors and compared them to the present sensor for AA detection. The developed sensor (Gel-g-PS/SPE) demonstrated a lower detection limit value than those reported in the literature.

**Table 2:** Analytical performances of the Gel-g-PS/SPE sensor compared with sensors reported in literature toward ascorbic acid detection

Electrode materials	Linear range	Lower detection limit	Reference
Melanin-like nanoparticles/SPCE	29–286 nM	0.4 nM	47
CNTs-4ABA/Au-IDA electrode	0–600 μM	15 μM	48
CuS@PB/GCE	5–3875 μM	0.240 μM	49
MoS_2_–O Cu/GCE	0.015–11.75 mM	22.2 nM	50
NiFe_2_O_4_/SPE	0.5–100 μM	10 μM	51
Branch-trunk Ag hierarchical nanostructures/GCE	0.017–1.8 mM	6.0 nM	52
NiCoO_2_/C modified GCE	10–2.63 mM	0.5 μM	53
PdNi/C/GCE	0.01–1.8 mM	0.5 μM	54
CeO_2_ NP/GC	1–500 μM	5.0 μM	55
l-Cysteine sonogel-carbon	0.05–1.0 mM	0.05 mM	56
Poly-trypan blue	1.0–630 μM	0.1 μM	57
Thioglycolate	1–500 μM	0.2 μM	58
Amino benzene sulfonic acid	35–175 μM	7.5 μM	59
Molecular imprinted polymer PAN	0.05–0.4 mM	18.0 μM	60
PAN-naphthalene sulfonic acid	5.0–60 mM	12.9 μM	61
Pan/SWi12/TiO_2_–MoO_3_	0.95–6.9 mM	1.2 μM	62
Tm_2_O_3_/ITO	0.2–8 mM	0.42 mM	63
Gel-g-PS/SPE	0.2–600 ppb	0.03 ppb	This work

### Interferences study

**Fig. 9:**
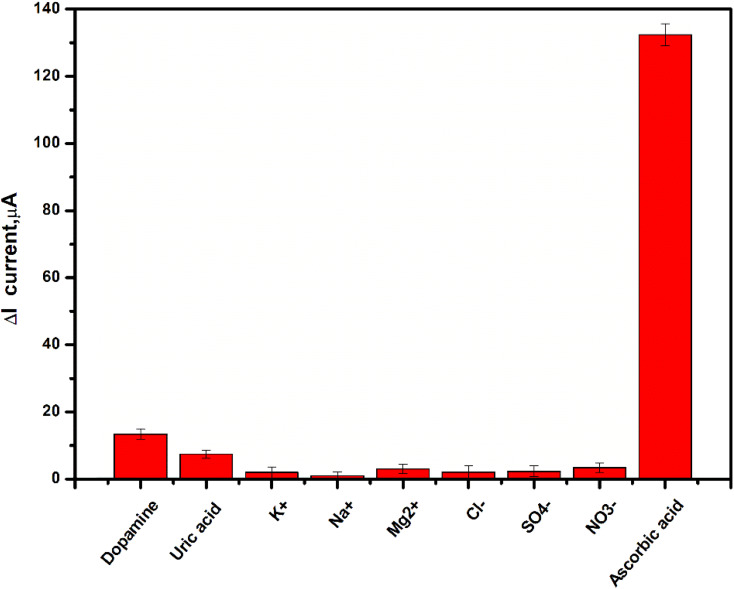
Interference study of the proposed sensor (Gel-g-PS/SPE) toward 60 ppb ascorbic acid and 120 ppb of uric acid, dopamine, K^+^, Na^+^, Mg^2+^, Cl^−^, SO^4−^, and NO^3−^ in PBS (pH 7.4). The experiments for each interferent were conducted in triplicate.

The selectivity study is the most significant examination for sensors. Therefore, the effect of interferent ions and substances (such as uric acid, dopamine, K^+^, Na^+^, Mg^2+^, Cl^−^, SO_4_^2−^, and NO_3_^−^) on the performance of Gel-g-PS/SPE towards AA detection was tested using the DPV technique. The current response of Gel-g-PS/SPE towards 60 ppb of AA and 120 ppb of different interferents was presented in the histogram of Fig. 9. A slight increase in the current value can be detected for only dopamine and uric acid, with a very high current response obtained for AA. Therefore, the Gel-g-PS/SPE displayed an excellent selectivity toward AA in the presence of interferents found in complicated samples such as human saliva or serum.

### Reproducibility, repeatability, and stability study of the proposed sensor

Reproducibility, repeatability, and stability are significant features of sensors, and were studied using the DPV technique. Reproducibility and repeatability studies of the Gel-g-PS/SPE were performed in a solution containing PBS (pH 7.4) with 100 ppb of AA using the DPV technique. For the reproducibility measurement, three independent Gel-g-PS modified electrodes were used for AA response, yielding results with a rogue system detection (RSD) of 5.3%. Moreover, the repeatability study involved five measurements of the same electrode, resulting in an RSD of 5.4%, revealing good repeatability. In the Gel-g-PS/SPE stability test, the electrode was stored at −4 °C in PBS (pH 7.4) and tested over a period of 1 month. The proposed sensor retained 96.7% of its initial sensitivity indicating good stability over the long term.

### Application of detection in fresh fruit juices and vitamin C tablet

For practical applications, the amount of ascorbic acid in fresh fruit juices (lemon, orange, and mango) and vitamin C tablets was measured by the DPV technique and Gel-g-PS/SPE. A standard addition method was applied for AA detection in the real samples. The peak current measurements performed by the DPV technique were compared with the calibration linear plot (*I*_pa_ against concentration presented in Fig. 8) to accurately determine the concentration of AA. The good recovery of the samples, as presented in Table 3, confirmed the successful applicability of the Gel-g-PS/SPE DPV measurements for AA detection in drug tablets and food samples.

Determination of ascorbic acid in (I) natural fruit juices and (II) pharmaceutical drug tablets

**Table:** 

(I)				
Sample (fruits)	Detected DPV mg/100 ml (*A*)	Added mg/100 ml (*B*)	Detected after addition (*C*)	Recovery % [(*C* − *A*)/*B*] × 100
Lemon	25.2	20	45.7	98.6
Orange	26.4	20	46.9	99.8
Mango	47.6	20	67.9	98.9

**Table:** 

(II)			
Sample	Ascorbic acid (mg/tablet)	Detected DPV	Recovery %
Vitamin C tablet			
	100	98.5	98.4
	150	147.2	98.3
	200	195.6	98.4

## Conclusion

Ascorbic acid (AA) detection is highly desirable for the pharmaceutical, cosmetics, and food industries. Therefore, it is crucial to develop an excellent performance functionalized hydrogel for effective AA detection. Regarding this, an inventive novel gelatin sulfonamide was created using a free radical-mediated grafting polymerization approach for electrochemical detection of AA in various fruits and pharmaceutical drug samples. First, the newly synthesized polymeric material was characterized, and its electrochemical properties on the Gel-g-PS/SPE for AA detection were investigated using cyclic voltammetry (CV) and differential pulse voltammetry (DPV) studies. An oxidation peak for AA was clearly observed on the Gel-g-PS/SPE surface with linear responses in the oxidation peak currents. A correlation coefficient of 0.98 and 0.977 was obtained for the linear ranges from 0.2–5 ppb and 20–600 ppb, with detection limit of 0.03 ppb. The proposed method demonstrated good selectivity, stability, reproducibility, and repeatability as well as excellent recovery rates in various fruits and pharmaceutical samples.

## Data availability

The authors confirm that the data supporting the findings of this study are available within the article.

## Author contributions

M. S. Hashem: conceptualization, writing – review & editing, visualization, formal analysis, investigation, data curation. Asmaa M. Fahim: formal analysis, investigation, data curation. Hend S. Magar: conceptualization, writing – review & editing, visualization, formal analysis, investigation, data curation.

## Conflicts of interest

The authors state that none of their known financial conflicts or interpersonal connections could have influenced the work that was published in this paper.
